# Patient risk stratification and tailored clinical management of post‐transplant CMV‐, EBV‐, and BKV‐infections by monitoring virus‐specific T‐cell immunity

**DOI:** 10.1002/jha2.175

**Published:** 2021-06-01

**Authors:** Anastasia Papadopoulou, Kiriakos Koukoulias, Maria Alvanou, Vassilios K. Papadopoulos, Zoe Bousiou, Vasiliki Kalaitzidou, Fotini S. Kika, Apostolia Papalexandri, Despina Mallouri, Ioannis Batsis, Ioanna Sakellari, Achilles Anagnostopoulos, Evangelia Yannaki

**Affiliations:** ^1^ Hematology Department‐Hematopoietic Cell Transplantation Unit, Gene and Cell Therapy Center “George Papanikolaou” Hospital Thessaloniki Greece; ^2^ Department of Genetics, Development and Molecular Biology, School of Biology Aristotle University of Thessaloniki Thessaloniki Greece; ^3^ Blood Bank Department General Hospital of Pella‐Giannitsa Giannitsa Greece; ^4^ Department of Medicine University of Washington Seattle Washington USA

**Keywords:** allogeneic hematopoietic cell transplantation, antithymocyte globulin, graft versus host disease, post‐transplant infections, virus‐specific T‐cells

## Abstract

**Background:**

Despite routine post‐transplant viral monitoring and pre‐emptive therapy, viral infections remain a major cause of allogeneic hematopoietic cell transplantation‐related morbidity and mortality.

**Objective:**

We here aimed to prospectively assess the kinetics and the magnitude of cytomegalovirus‐(CMV), Epstein Barr virus‐(EBV), and BK virus‐(BKV)‐specific T cell responses post‐transplant and evaluate their role in guiding therapeutic decisions by patient risk‐stratification.

**Study design:**

The tri‐virus‐specific immune recovery was assessed by Elispot, in 50 consecutively transplanted patients, on days +20, +30, +60, +100, +150, +200 post‐transplant and in case of reactivation, weekly for 1 month.

**Results:**

The great majority of the patients experienced at least one reactivation, while over 40% of them developed multiple reactivations from more than one of the tested viruses, especially those transplanted from matched or mismatched unrelated donors. The early reconstitution of virus‐specific immunity (day +20), favorably correlated with transplant outcomes. Εxpanding levels of CMV‐, EBV‐, and BKV‐specific T cells (VSTs) post‐reactivation coincided with decreasing viral load and control of infection. Certain cut‐offs of absolute VST numbers or net VST cell expansion post‐reactivation were determined, above which, patients with CMV or BKV reactivation had >90% probability of complete response (CR).

**Conclusion:**

Immune monitoring of virus‐specific T‐cell reconstitution post‐transplant may allow risk‐stratification of virus reactivating patients and enable patient‐tailored treatment. The identification of individuals with high probability of CR will minimize unnecessary overtreatment and drug‐associated toxicity while allowing candidates for pre‐emptive intervention with adoptive transfer of VSTs to be appropriately selected.

## 1 INTRODUCTION

Allogeneic hematopoietic cell transplantation (allo‐HCT) is a potentially curative treatment for hematological disorders. Among its main hurdles however, is the profound T‐cell deficiency, resulting in development of viral infections ‐ most commonly by cytomegalovirus (CMV), Epstein Barr virus (EBV) and polyomavirus type I (BKV)‐, and the substantial transplant‐related morbidity and mortality [1, 2]. The reconstitution of antiviral immunity post‐allo‐HCT is often delayed or/and severely impaired by the immunosuppression administered to prevent or treat the immunological complications of allogeneic transplantation, affecting both the quantity and the quality of virus‐specific T‐cells (VSTs) [3]. The importance of a specific and robust anti‐viral immune reconstitution is emphasized by the fact that strategies boosting antiviral immunity, such as immunotherapy with donor‐ or third‐party‐derived VSTs, have provided protection with 70–90% response rates [1, 4–7].

Heretofore, clinicians relied exclusively on the viral load monitoring, to guide interventions in treating viral infections post‐transplant. Given that immunity is a dynamic process, close monitoring of VST immune reconstitution (VST‐IR), may identify patients able to potentially self‐control viral reactivations and, eventually, provide the basis for tailored management of antiviral therapy to those in real need avoiding unnecessary overtreatment or/and the outgrowth of drug resistant viral variants.

As opposed to single‐virus‐specific immune reconstitution, the recovery of viral immunity against multiple viruses has been limitedly investigated in HCT [8, 9]. We here aimed, by concomitant monitoring of IR against the most common viruses post‐allo‐HCT, namely CMV, EBV, and BKV, to prospectively recognize correlations between viral reactivation and the timing and kinetics of VST‐IR, compare the recovery of functional VSTs with virological outcomes and provide surrogate markers for identifying patients able to successfully clear the infection and fine‐tuning the clinical‐decision making.

## 2 MATERIALS AND METHODS

### 2.1 Subjects

Fifty consecutive allo‐HCT patients were included in this prospective study, approved by the Institutional Review Board of the George Papanikolaou Hospital and performed in accordance with the Declaration of Helsinki.

### 2.2 Virological monitoring

CMV and EBV viral loads were routinely monitored by quantitative PCR (Qiagen) on blood samples, once a week. BKV load was measured by quantitative PCR (Geneproof) in urine every other week at the presence of clinical signs/symptoms or/and at request of the treating physician.

### 2.3 Definitions

CMV/EBV reactivation/infection was defined as increasing viremia with >500 copies/mL in two consecutive measurements or > 1000 copies/mL in a single screenshot. BKV reactivation/infection was defined as viruria with >10^7^ copies/mL. Virus detection in tissue fluid or sample accompanied by clinical symptoms was defined as viral disease.

Virus reactivating patients were classified as (i) complete responders (CRs) if there was a return of viral load to normal range and resolution of clinical signs/symptoms, (ii) partial responders (PRs) if there was at least 50% decrease in viral load from baseline or 50% clinical improvement, (iii) non‐responders (NRs) when changes were insufficient to qualify as CR or PR.

### 2.4 Immunological monitoring by enzyme‐linked immunospot

Immunological monitoring was performed in peripheral blood samples collected at days +20, +30, +60, +100, +150, +200 post‐allo‐HSCT and in case of viral reactivation, weekly for 1 month, as shown in Figure S1. Peripheral blood mononuclear cells (PBMCs) were stimulated with peptides spanning CMV and EBV antigens whereas BKV‐STs were firstly expanded in culture (Supplementary Information) [4, 5, 10], due to their low frequency in blood. PBMCs or expanded BKV‐STs were pulsed with viral peptides (CMV: IE1, pp65; EBV: EBNA1, LMP2, BZLF1; BKV: LT, VP1; JPT Peptide Technologies), and IFN‐γ secretion was measured by enzyme‐linked immunospot (Mabtech). Spot‐forming cells (SFCs) were counted on Eli.Scan (A.EL.VIS) using Eli.Analyse software V6.2.SFC. Response, expressed as SFCs per input cells, was considered specific if total SFCs against viral antigens were ≥30/5 × 10^5^ PBMCs or 2 × 10^5^ BKV‐expanded cells.

### 2.5 Statistical analysis

Analysis of viral reactivations according to donor type was based on count methods, since each patient could have more than one reactivation events. Matched sibling donors (MSDs) used as the reference category and for each donor sub‐group, incidence rates (IR) were calculated as total number of events per 10,000 person‐days (equivalent to "per 100 patients per 100 days") of observation. Byar's approximation method was used for the IR confidence intervals (CIs). Differences between groups were assessed with IR Ratios (CIs according to Wald method) and chi‐square tests, calculated using median‐unbiased estimation and exact methods (mid‐*p*). This type of analysis does not take into account the timing of the events, (whether they occur earlier or later during the follow‐up) but only the total count of observed events. Analysis was performed in R programming environment with epitools v0.5–10.1 package.

In order to identify factors associated with VSTs, univariate analysis was performed with non‐parametric methods (Kruskal‐Wallis tests) because their numbers were not normally distributed. Multivariate analysis was performed with multiple linear regression model; VSTs were log‐transformed, in order to meet the model's assumptions.

Non‐relapse mortality (NRM) and timing of reactivation events were analyzed with survival methods. Cumulative incidence of reactivation and NRM were calculated with the competing risk model, where death or death from other causes, respectively, was considered a competing event. Comparisons between groups were made with Gray's test, a modified chi‐square test, while survival curves were drawn according to Kaplan‐Meier method. Analyses and curves were performed in the R programming environment (v3.6.1) with "survival" and "cmprsk" statistical packages.

To assess whether CR of viral reactivation was correlated with the VST kinetics and could be predicted based on certain cut‐offs VST numbers or of the net increase of VSTs from the onset of reactivation up to 2 weeks later (Delta‐SFC [ΔSFC:max‐VSTs minus onset‐VSTs]), a receiver operating characteristic (ROC)‐curve analysis was performed. The reactivating patients’ cohort was then split at the optimal VST cut‐off, and cumulative incidences of CR between the above or below cut‐off sub‐cohorts were compared with the competing risk model.

Data are presented as mean ± SEM or median (range) when values follow normal or abnormal distribution, respectively. ANOVA followed by Tukey's test or a nonparametric Mann‐Whitney U test were used for analysis of differences between data sets or multiple comparisons, respectively.

## 3 RESULTS

### 3.1 Patient characteristics

Fifty adult patients, who consecutively received allo‐HCT, from MSD, matched unrelated donor (MUD), mismatched unrelated donor (MMUD), and haploidentical donor (haplo), were enrolled in the study. Haplo‐transplant recipients receiving other than unmanipulated bone marrow cells and post‐transplant Cy (PTCy) were not included in the study (*n* = 2). Acute graft‐versus‐host‐disease prophylaxis included antithymocyte globulin (ATG), PTCy, Cyclosporin/Methotrexate, Mycophenolate Mofetil; due to ATG's dominant immunomodulating effect, patients were classified as receiving or not, ATG (Table 1). Viral prophylaxis in all patients included administration of acyclovir/valacyclovir (letermovir as primary anti‐CMV prophylaxis became available later during the study). Pre‐emptive treatment for CMV and EBV antigenemia included ganciclovir or foscarnet and rituximab, respectively. BKV hemorrhagic cystitis was treated with cidofovir. All patients received anti‐viral treatment, except otherwise indicated.

**TABLE 1:** Clinical characteristics of the study population

Patients, *n*	50
Age, median years (range)	40.5 (17–69)
Sex, *n* (%)	
Male	30 (60)
Female	20 (40)
Diagnosis, *n* (%)	
Acute myeloblastic leukemia	21 (42)
Acute lymphoblastic leukemia	13 (26)
Myelodysplastic syndrome	5 (10)
Myeloproliferative neoplasms	4 (8)
Hodgkin lymphoma	3 (6)
Chronic lymphocytic leukemia (CLL)	2 (4)
Non‐Hodgkin lymphoma	1 (2)
Multiple myeloma	1 (2)
Conditioning regimen, *n* (%)	
Myeloablative	38 (76)
RIC	12 (24)
Donor type, *n* (%)	
Matched sibling donor	17 (34)
Matched unrelated donor	20 (40)
Mismatched unrelated donor	8 (16)
Haploidentical	5 (10)
Stem cell source, *n* (%)	
Bone marrow	5 (10)
Peripheral blood stem cells	45 (90)
Donor/recipient CMV serostatus, *n* (%)	
Positive/positive (D+/R+)	28 (56)
Negative/positive (D‐/R+)	7 (14)
Positive/negative (D+/R‐)	7 (14)
Negative/negative (D‐/R‐)	6 (12)
Unknown	2 (4)
Donor/recipient EBV serostatus, n (%)	
Positive/positive (D+/R+)	34 (68)
Negative/positive (D‐/R+)	5 (10)
Positive/negative (D+/R‐)	4 (8)
Negative/negative (D‐/R‐)	3 (6)
Unknown	4 (8)
Acute GvHD, n (%)	
grade 0–I	38 (76)
grade II–IV	12 (24)
ATG‐based GvHD prophylaxis or treatment, *n* (%)	
ATG	35 (70)
No ATG	15 (30)

Abbreviations: ATG, anti‐thymocyte globulin; CMV, cytomegalovirus; EBV, Epstein‐Barr virus; GvHD, graft‐versus‐host disease GvHD; RIC, reduced intensity conditioning.

### 3.2 Viral episodes, IRs, and association with transplant outcomes

From the 50 study subjects, 37 developed at least one viral reactivation from CMV, EBV, and BKV, reaching a total of 78 reactivations (28, 33, 17 CMV, EBV, BKV infections, respectively) through 200 days post‐HCT. Fourteen reactivations were diagnosed as or progressed to viral disease (CMV:2 [retinitis, pneumonitis], EBV:1 [encephalitis], BKV:11 [hemorrhagic cystitis]; Table S1), from which one (EBV encephalitis) led to death. The median time to reactivation from any of the three viruses was 35 days (CMV: 37 days [−2 −164], EBV: 28 days [7–196], BKV: 47 days [21–115]). The highest incidence of viral reactivations (67%) was observed by day 30 post‐transplant (22%, 35%, 10% for CMV, EBV, BKV, respectively), while the overall incidence substantially declined by days 100 (20%) and 200 (10%) (Figure S2). The decrease in the incidence of viral reactivations over time, was inversely correlated with VSTs reconstitution (Figure S3).

**FIGURE 1:**
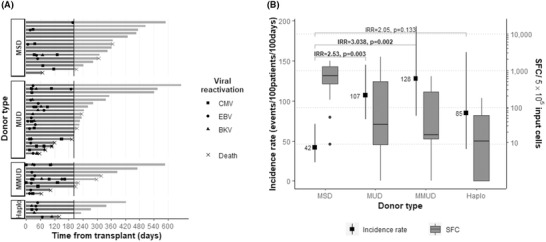
Viral reactivations (A) per donor group. Each bar represents the observation time per patient, grouped by donor type. Occurrence of viral reactivation up to day 200 (squares: CMV, circles: EBV, triangles: BKV) or death (‘x’) is marked on the bar. (B) Per donor group in accordance with early (day 20) virus‐specific immune reconstitution. The squares with whiskers represent incidence rate of infections from all viruses per type of donor (95% confidence intervals). Incidence rate ratios and *p*‐values were based on exact mid‐*p* methods. The boxplots depict the distribution of total VSTs on day 20 post‐allo‐HCT for each type of transplant. Outliers are shown as dots Abbreviations: BKV, BK virus; CMV, cytomegalovirus; EBV, Epstein Barr virus; Haplo, haploidentical donor; MMUD, mismatched unrelated donor; MSD, matched sibling donor; MUD, matched unrelated donor; SFC, spot forming cells.

More than 40% of the patients (21/50) presented multiple reactivations, with a median of two (1–6) reactivations per affected patient (Figure 1A). The MSD‐group experienced less infectious episodes over MUD (*p* < 0.003), MMUD (*p* = 0.002), and haplo (*p* = 0.13) presenting the lowest IR ratio (95% CI, 1 vs 2.5 vs 3 vs 2, respectively) (Figure 1B; S4 Table S2). The lower overall infection rate in the MSD group coincided with earlier VST‐IR over the other donor groups (Figure 1Β; S5).

CMV seropositive recipients demonstrated increased (45.7% vs 23.1%), albeit not statistically significant, incidence of CMV reactivations (*p* = 0.15) over the seronegative hosts, given the constraints of a relatively small number of patients assessed in each group (Table S3). By looking at the kinetics of VSTs, at different time points and in association with the recipient serostatus, we observed significantly earlier and higher CMV‐specific immune reconstitution between days +30 and +100 in seropositive over seronegative hosts, probably reflecting boost immune responses to the increased rate of reactivations in seropositive patients (Figure S6). Similar, albeit not significant, kinetics of EBV‐ST reconstitution was observed in EBV seropositive recipients (Table S3, Figure S6).

**FIGURE 2:**
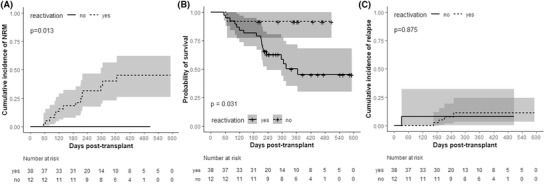
Cumulative incidence of non‐relapse mortality (A), overall survival (B), and relapse (C) according to reactivation status (Kaplan‐Meier curves with 95% confidence intervals [grey zones], *p*‐value derived from Cox proportional hazards’ model (B) or modified Cox subdistribution hazards model (A, C))

At a median follow‐up of 284 days (range 56–592), the NRM was 30% and significantly associated with the occurrence of viral reactivations; 15 of 38 patients (40%) who developed at least one reactivation succumbed to transplant complications, whereas none of the 12 (0%) non‐reactivating patients died from other than relapse causes (*p* = 0.01; Figure 2A). The higher NRM among virus reactivating over virus non‐reactivating patients resulted in lower OS (52.7%, Δm 12 months vs 91.7%, Δm not reached, *p* = 0.03) (Figure 2B). Viral reactivations had a limited impact on relapse rates (≥1 reactivation vs no reactivation: 10.5% vs 8.3%*, p = ns;* Figure 2C).

### 3.3 Viral reactivation and VST‐IR

**FIGURE 3:**
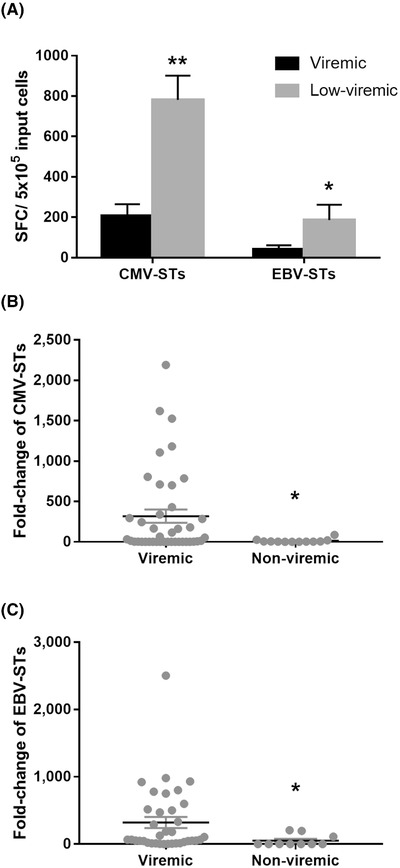
VSTs and viral reactivations. (A) CMV‐ST and EBV‐ST levels at the onset of reactivations in patients who experienced CMV and EBV DNAemia without reaching the threshold values for receiving treatment (low‐viremic patients, grey bars) and in patients who ultimately developed CMV and EBV infection (viremic, black bars). Bars represent the mean value within each group, and error bars are the standard error of the mean. Differences between data sets were analyzed using Mann‐Whitney test (**p = 0.0016 and *p = 0.0207). (B and C) CMV‐ST (B) and EBV‐ST (C) expansion in individual patients who developed viral reactivations (infection or low viremia) relative to those subjects who did not (non‐viremic). Each dot represents one patient, and the lines represent the mean value within each group and the standard error of the mean. Differences between data sets were analyzed using unpaired one‐tailed *t*‐test (*p > 0.05) Abbreviations: CMV‐STs, CMV‐specific T cells; EBV‐STs, EBV‐specific T cells; SFC, spot‐forming cells; VSTs, virus‐specific T cells.

To better understand the temporal relationship between viral reactivations and VST‐IR, we compared the CMV‐ and EBV‐ST levels at reactivation onset between recipients who developed infection as per the definition and “low‐viremic” patients who experienced CMV and EBV DNAemia without reaching the threshold values above which viremia was considered infection and treated. The low‐viremic patients had significantly higher numbers of cytokine‐producing VSTs at reactivation onset than those who ultimately developed viral infection (Figure 3A), suggesting that functional VSTs protected patients against the development of relevant infections. Notably, VSTs markedly expanded from baseline in all viremic patients relative to non‐viremic subjects (Figures 3B and 3C), suggesting that antigen stimulation triggers VST proliferation.

### 3.4 Risk factors for impaired VST‐IR

**FIGURE 4:**
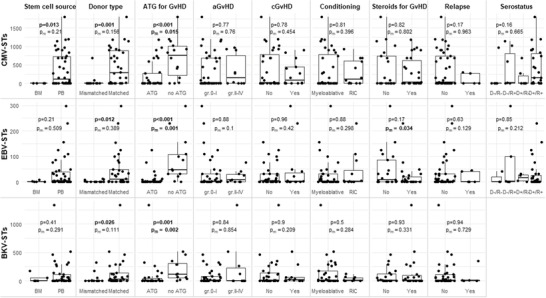
Risk factors for impaired virus‐specific immunity. Virus‐specific T‐cells on day 20–30, according to categorical variables. Distribution of VSTs is shown as boxplot/dot plot combination. *p*‐values for univariate analysis (*p*) are calculated with Kruskal‐Wallis non‐parametric statistics. Multivariate *p*‐values (p_m_) are calculated with multiple linear regression on the log‐transformed VST values. Significant differences are indicated in bold. Key: donor type; matched (sibling/unrelated) versus mismatched (unrelated/haplo); *multivariate analysis’ *p*‐values estimate differences from reference category (both seronegative) Abbreviations: aGvHD, acute GvHD; ATG, anti‐thymocyte globulin; BKV‐STs, BK virus‐specific T cells; CMV‐STs, cytomegalovirus‐specific T cells; cGvHD, chronic GvHD; D, donor; EBV‐STs, Epstein Barr virus‐specific T cells; GvHD, graft‐versus‐host‐disease; R, recipient; RIC, reduced intensity conditioning.

To identify risk factors for delayed or/and impaired VST‐IR, the VST levels at an early time point (day +20), were checked against different variables in univariate and multivariate analysis. ATG administration was strongly associated with significantly lower CMV‐, EBV‐, and BKV‐ST levels both in the univariate and multivariate analysis whereas donor type (mismatched vs matched) did not retain significance in the multivariate analysis (Figure 4). Notably, steroid administration raised as a contributing factor toward delayed reconstitution of EBV‐specific T‐cell immunity only in the multivariate analysis, probably due to a confounding effect of the other covariates in the univariate analysis.

### 3.5 VST‐IR and clinical outcome

**FIGURE 5:**
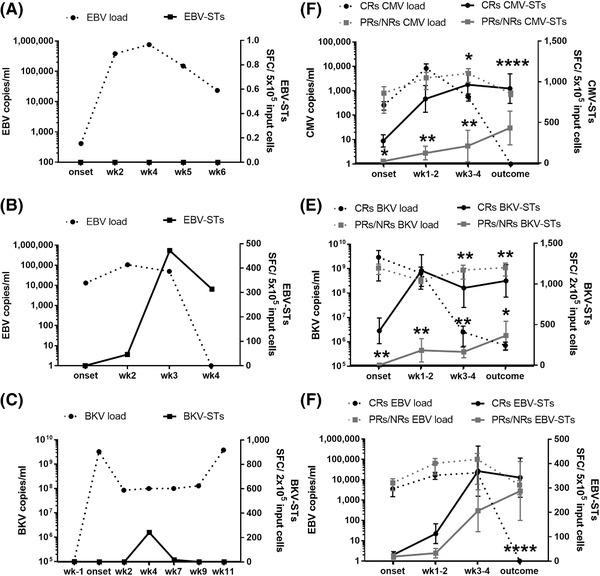
Successful viral control associated with robust VST recovery. (A‐C) Individual patient graphs of viral load and VST reconstitution. (A) Example of a patient who reactivated EBV but failed to develop EBV‐STs and succumbed to EBV encephalitis. (B) Example of a subject who effectively controlled EBV after reactivation and subsequent expansion of EBV‐STs. (C) Example of a subject who reactivated BKV, did not develop sustained numbers of BKV‐STs, and suffered from severe, painful, and persistent hemorrhagic cystitis. Dotted lines and solid lines illustrate viral loads and VSTs, respectively. (D‐F) Expansion of CMV‐STs (D), BKV‐STs (E), and EBV‐STs (F) in association with viral loads between CRs and PRs/NRs. Dotted lines illustrate viral loads and solid lines illustrate VSTs. Black lines illustrate CRs, and grey lines illustrate PRs/NRs. Differences between data sets were analyzed using Mann‐Whitney test (*p < 0.043, **p < 0.006, and ****p < 0.0001) Abbreviations: BKV‐STs, BKV‐specific T cells; CMV‐STs, CMV‐specific T cells; CRs, complete responders; EBV‐STs, EBV‐specific T cells; PRs/NRs, partial and non‐responders; SFC, spot‐forming cells; wk, week.

VST‐IR correlated with the clinical outcome; the majority of patients not developing VSTs failed to control infection, while patients with VST rebounds either cleared the infection or developed only low viremia. This positive correlation between VST‐IR and successful viral control after allo‐HCT and vice versa is shown in three representative cases. The first patient reactivated EBV but did not develop detectable EBV‐specific response and succumbed to EBV encephalitis despite receiving rituximab. The second, who rapidly amplified EBV‐STs, effectively controlled the viral burden. The third patient who reactivated BKV and did not develop sustained presence of BKV‐STs, suffered from prolonged hemorrhagic cystitis despite cidofovir treatment (Figures 5A‐5C). To better understand the role of VSTs in controlling viral reactivations, reactivating patients were classified based on their response. As regards CMV, CRs presented a massive CMV‐ST expansion, followed by a dramatic decrease in viral load and effective control of the reactivation/disease (Figure 5D). In contrast, at reactivation onset, partial and no responders (PRs/NRs) had significantly lower CMV‐STs which either did not expand or profoundly delayed to rebound over CRs (*p* = 0.039), ultimately being unable to control CMV. Likewise, among patients with BKV reactivations, CRs had higher VST numbers at reactivation onset (*p* = 0.0031) which coincided with strong T‐cell responses leading to resolution of BKV replication and shedding, in contrast to PRs/NRs where the limited or delayed recovery of BKV‐STs couldn't control the increasing viral burden (Figure 5E). A similar, albeit less distinct, correlation was observed in patients with EBV reactivations (Figure 5F). When subgrouping only those patients who developed viral diseases (*n* = 14 in total, Table S1), again CRs (*n* = 9) presented higher median VST numbers at reactivation onset and reached higher VST peaks (Δm 100 SFC /2–5 × 10^5^ cells [0–1222] and Δm 1,238 SFC/2‐5 × 10^5^ cells [323–1700], respectively) over PR/NRs (*n* = 5) (Δm 0 SFC /2‐5 × 10^5^ cells [0–22] and Δm 242 SFC/2‐5 × 10^5^ cells [65–1377]) (*p* = 0.1 and *p* = 0.04, respectively) (Figure S7).

### 3.6 Predicting CRs and guiding therapeutic decision based on certain levels of virus‐specific immunity

**FIGURE 6:**
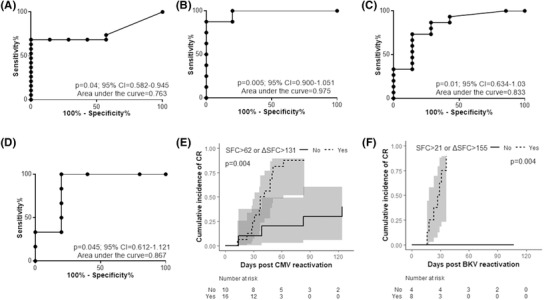
Receiver operating characteristic (ROC) curves of CMV‐ and BKV‐ST levels displaying as outcome the complete response. (A and B) ROC curves of the number of CMV‐STs (A) and BKV‐STs (B) at reactivation, displaying complete response as the outcome. (C and D) ROC curves of the ΔSFC of CMV‐STs (C) and BKV‐STs (D) 2 weeks post‐reactivation, displaying complete response as the outcome. (E and F) Cumulative incidence of complete response according to the predictive for CR cut‐offs of CMV‐ST and BKV‐ST absolute SFCs at reactivation (E) or their ΔSFC post‐reactivation (F) in comparison with the combo variable of both SFCs and ΔSFC below cut‐off values. The grey zones represent confidence intervals of 95%

To determine a threshold for protective VST immunity, thus avoiding overtreatment or stratifying patients to different therapy options, we performed ROC analysis. In CRs, the absolute VST values at reactivation and the net increase (ΔSFC) of VSTs from the onset of reactivation up to 2 weeks later (ΔSFC:max‐VSTs minus onset‐VSTs) were compared to their counterparts of PRs/NRs. A cutoff ≥ 63 and ≥ 22 SFC/2–5 × 10^5^ cells for CMV and BKV respectively, at time of reactivation, was found optimal, allowing discrimination with high sensitivity and specificity of patients who cleared the infection over PRs/NRs (*p* = 0.04, *p* = 0.005, respectively; Table 2, Figures 6A and 6B). Moreover, a ΔSFC ≥ 132 CMV and ≥ 156 BKV/2‐5 × 10^5^ cells, 1–2 weeks post‐reactivation was predictive with high sensitivity and specificity of a CR (*p* = 0.01, *p* ≤ 0.05, respectively; Table 2, Figures 6C and 6D). Importantly, CMV reactivating patients in whom either of the CR predictive criteria was fulfilled (SFCs ≥ 63 or ΔSFC ≥ 132) had 94% probability of CR as compared to only 40% probability of CR of those having both values below cut‐off (HR:4.96, 95% CI 1.42–17.31, *p* = 0.004) (Figure 6E). More strikingly, BKV reactivating patients reaching either one of the CR predictive cut‐offs, had 100% probability of CR as compared to 0% of patients having both SFCs ≤ 21 and ΔSFC ≤ 155 (*p* = 0.004; HR not provided due to small sample size, Figure 6F).

**TABLE 2:** Sensitivity and specificity of threshold levels of VSTs and their corresponding ΔSFC post reactivation

Value	Threshold	Sensitivity (%)	Specificity (%)	Optimal threshold
**CMV‐STs at reactivation**	>54.5	68.42	85.71	
	>62.5	68.42	100	>62.5 CMV‐STs
	>68	63.16	100	
**BKV‐STs at reactivation**	>3	87.5	80	
	>21	87.5	100	>21 BKV‐STs
	>47.5	75	100	
**CMV‐ST ΔSFC post‐reactivation**	>104	73.33	71.43	
	>131.5	73.33	85.71	>131.5 CMV‐ST ΔSFC
	>157.5	66.67	85.71	
**BKV‐ST ΔSFC post‐reactivation**	>43.5	100	60	
	>155.5	100	80	>155.5 BKV‐ST ΔSFC
	>263.5	83.33	80	

Abbreviations: BKV‐STs, BK virus‐specific T cells; CMV‐STs, cytomegalovirus‐specific T cells.

Although ROC analysis did not provide predictive values for EBV‐specific‐IR (Figures S8A and S8B), a rapid and considerable EBV‐ST expansion during the initial phase of EBV reactivation was suggestive of subsequent endogenous viral control. Indeed, we used EBV‐ST expansion as a tool for clinical decision making and patient‐tailored management, in three EBV‐reactivating patients in whom the EBV‐ST levels and viral load were closely monitored from the onset of viral reactivation onwards. Based on a rapid and significant EBV‐ST expansion during the initial phase of reactivation (median ΔSFC 2 weeks post‐reactivation: 365 SFC/5 × 10^5^ cells, range: 107–855), those patients remained under close monitoring without receiving antiviral pharmacotherapy. Notably, in all patients, EBV reactivation was effectively controlled by the spontaneously expanded endogenous EBV‐STs without any treatment (representatively depicted in Figure S8C).

## 4 DISCUSSION

Despite improvements in anti‐viral pharmacotherapy, CMV, EBV, and BKV reactivations remain a leading cause of morbidity and mortality following allo‐HCT [1, 2]. Unidimensional guidance of prophylactic, pre‐emptive, and therapeutic interventions in transplant recipients by monitoring the viral load without also considering the status of virus‐specific immunity, often results in unnecessary treatment, treatment‐related toxicities, or/and emergence of drug resistance. On the other hand, a diagnostic tool to safely predict the presence of functional cellular immunity and thus the subsequent successful control of viral infections remains elusive. We here, prospectively focused on the timing, kinetics, and magnitude of VST‐IR to common post‐transplant viruses (CMV, EBV, and BKV), as a means to risk‐stratify patients with viral reactivations and fine‐tuning the clinical‐decision making.

In total, 74% of patients developed CMV or/and EBV or/and BKV reactivations, with a median of two viral episodes/patient. Apart from the substantial morbidity, and in agreement with other reports [11, 12, 13, 14, 15, 16, 17], viral reactivations from these three viruses, correlated also with adverse outcomes as regards OS and NRM, thus underscoring the magnitude of the indirect mortality induced by viral infections and their treatment. The huge human and financial cost associated with the management of viral infections post‐transplant provides both a scientific and economic rationale for patient risk‐stratification, so as to avoid unnecessary treatment in those patients who have acquired functional virus‐specific immunity or to proceed with interventions such as VST immunotherapy for those at high‐risk.

Although the correlation between VST presence and viral control has been demonstrated post‐solid organ transplantation for all three viruses [18, 19, 20, 21, 22, 23, 24, 25, 26, 27], reports in the HCT setting mostly have focused on CMV [16, 28, 29, 30, 31, 32, 33, 34, 35, 36, 37, 38, 39, 40, 41, 42, 43, 44, 45, 46, 47, 48, 49, 50, 51, 52, 53, 54, 55, 56]. In our study, by simultaneously monitoring functional T‐cell immunity against a broad spectrum of clinically problematic viruses after allo‐HCT, in conjunction with viral load, we showed that VST rebounds, even in patients with viral diseases, were associated with marked reductions of the relevant viral loads and a favorable clinical outcome. Further supporting that even subclinical levels of viral replication boost antigen‐experienced T‐cell reconstitution [8, 28], viral reactivating patients (infected or low viremic) demonstrated considerable VST expansion from baseline relative to non‐viremic subjects.

The VST‐IR was serially measured by the levels of CD3+ CMV‐, EBV‐, and BKV‐STs in PBMCs. Others have proposed monitoring of CD8+ CMV‐STs as a prognostic tool to identify allo‐HCT patients at high risk for CMV‐infections [52, 57], however, by evaluating only CD8+ cells, and given the importance of CD4+ STs in controlling infections [58], the clinical response may be misinterpreted or/and underestimated.

Hematopoietic stem cell transplant physicians will more accurately guide their therapeutic decisions if immune competence against viruses could be precisely estimated, enabling the discrimination of patients with solid antiviral immunity and high probability of CR from those having low probability of clearing the infection and being dependent on pre‐emptive interventions. Several groups, focusing on CMV, have tried to identify VST thresholds predictive of protection from viral reactivation [59, 60, 61, 62]. Instead, we here provide, a prediction model that could identify with at least 94% probability, among CMV and BKV reactivating patients, those who could successfully clear the infection, based on certain VST levels at reactivation or their max ΔSFC 1–2 weeks later. Although ROC analysis couldn't provide CR predictive cut‐offs in EBV reactivating patients, three patients for whom high EBV‐VSTs at reactivation were detected, and a considerable expansion was measured in the following 2 weeks, cleared EBV infection without receiving rituximab.

Notwithstanding the need for validation of the predictive cut‐offs for viral complete response in a larger, multicenter study, our data stress out the importance of monitoring VST‐IR by functional assays that can be used to risk‐stratify transplanted patients with viral infections and guide their clinical management. The identification of patients at low risk for morbidity and mortality from viral reactivations will minimize unnecessary overtreatment and drug‐associated toxicity. On the other hand, identification of patients at‐risk might lead to pre‐emptive intervention with intense antiviral pharmacotherapy or virus‐specific immunotherapy. Guided pre‐emptive therapeutic choices based on the actual individual risk for controlling viral infections will potentially result in lower morbidity and better survival chances in allo‐HCT patients as well as substantially decrease the post‐transplant care cost.

## CONFLICT OF INTEREST

The authors declare that there is no conflict of interest that could be perceived as prejudicing the impartiality of the research reported.

## FINANCIAL INFORMATION

Funding for this project was provided in part by Research, Technology Development and Innovation (RTDI) State Aid Action “RESEARCH ‐ CREATE ‐ INNOVATE” and in part by a Research Grant award granted by the European Hematology Association.

## AUTHOR CONTRIBUTION

Conceptualization: Apostolia Papalexandri and Evangelia Yannaki. Methodology: Apostolia Papalexandri, Kiriakos Koukoulias, Maria Alvanou, Vassilios K. Papadopoulos, Zoe Bousiou, Vasiliki Kalaitzidou, and Anastasia Papadopoulou. Investigation: Apostolia Papalexandri, Kiriakos Koukoulias, Maria Alvanou, Vassilios K. Papadopoulos, Zoe Bousiou, Vasiliki Kalaitzidou, Fotini S. Kika, Anastasia Papadopoulou, Despina Mallouri, Ioannis Batsis, Ioanna Sakellari, and Evangelia Yannaki. Writing – original draft: Apostolia Papalexandri and Evangelia Yannaki. Writing – review and editing: Apostolia Papalexandri, Vassilios K. Papadopoulos, and Evangelia Yannaki. Funding acquisition: Apostolia Papalexandri, Achilles Anagnostopoulos, and Evangelia Yannaki. Resources: Achilles Anagnostopoulos and Evangelia Yannaki. Supervision: Achilles Anagnostopoulos and Evangelia Yannaki.

## Supporting information



Supporting Infomation
