# Supplementary material for: Patient risk stratification and tailored clinical management of post‐transplant CMV‐, EBV‐, and BKV‐infections by monitoring virus‐specific T‐cell immunity
Source: EJHaem. 2021 Jun 1;2(3):428–39. doi: 10.1002/jha2.175 (PMC9175754; doi:10.1002/jha2.175)
Supplement: Supplementary file 1 — Supporting Infomation [file JHA2-2-428-s001.pdf]

Supplementary Information

Supplementary tables

S1 Table. Patients with viral reactivation or disease.

	CMV	EBV	BKV
Viremia/viruria, n (%)			
Yes	19 (38)	31 (62)	16 (32)
No	31 (62)	19 (38)	34 (68)
Disease, n (%)			
Yes	2 (4)	1 (2)	11 (22)
No	48 (96)	49 (98)	39 (78)

Abbreviations: CMV, cytomegalovirus; EBV, Epstein-Barr virus; BKV, BK virus.

S2 Table

Virus	Donor type	Event counts	Person-days	Incidence Rate* (95% CI)	IRR (95% CI)	p-value†
CMV	MSD	6	2868	20.9 (8.7 – 43.1)	1	reference
	MUD	12	3557	33.7 (18.4 – 57.1)	1.6 (0.6- 4.7)	0.349
	MMUD	9	1565	57.7 (28.4 – 105)	2.7 (1 – 8.3)	0.058
	Haplo	1	939	10.6 (1 – 49.6)	0.6 (0 – 3.4)	0.591
EBV	MSD	5	2868	17.4 (6.6 – 38.2)	1	reference
	MUD	16	3557	45 (26.8 – 71.3)	2.5 (1 – 7.9)	0.055
	MMUD	8	1565	51.1 (24.1 – 96.5)	2.9 (0.9 – 9.9)	0.062
	Haplo	4	939	42.6 (14.2 – 101.3)	2.5 (0.6 – 9.6)	0.206
BKV	MSD	1	2868	3.5 (0.3 – 16.3)	1	reference
	MUD	10	3557	28.1 (14.4 – 49.9)	7.1 (1.4 – 177.4)	**0.016**
	MMUD	3	1565	19.2 (5.3 – 51.1)	5 (0.6 – 144.7)	0.145
	Haplo	3	939	31.9 (8.8 – 85.2)	8.4 (1 – 241.1)	0.053
Total	MSD	12	2868	41.8 (22.8 – 70.9)	1	reference
	MUD	38	3557	106.8 (76.8 – 145)	2.5 (1.4 – 5.1)	**0.003**
	MMUD	20	1565	127.8 (80.5 – 193.5)	3 (1.5 – 6.4)	**0.002**
	Haplo	8	939	85.2 (40.2 – 160.8)	2 (0.8 – 5)	0.133

* events per 10.000patient-days, IRR: incidence rate ratio, MSD was the reference category
† exact mid-p method

**S3 Table. Univariate analysis.**		**CMV reactivation (N,%)**		
		**No**	**Yes**	**p-value**
**CMV serostatus**	D+R+	15 (53,6)	13 (46,4)	0.52 (NS) (Pearson's chi-square)
	D-R+	4 (51,7)	3 (42,9)	
	D+R-	5 (71,4)	2 (28,6)	
	D-R-	5 (83,3)	1 (16,7)	
**CMV recipient serostatus**	R+	19 (54,3)	16 (45,7)	0.15 (NS) (Pearson's chi-square)
	R-	10 (76,9)	3 (23,1)	
**CMV-STs**	Mean	423,8	216,5	p=0.107 (t-test)
	Standard deviation	510	352,9	
	Median	155	8,5	p=0.301 (Mann-Whitney U-test)
	Interquartile range	819	375	
		**EBV reactivation (N, %)**		
		**No**	**Yes**	**p-value**
**EBV serostatus**	D+R+	12 (35,3)	22 (64,7)	0.97 (NS) (Pearson's chi-square)
	D-R+	2 (40)	3 (60)	
	D+R-	1 (25)	3 (75)	
	D-R-	1 (33,3)	2 (66,7)	
**EBV recipient serostatus**	R+	14 (35,9)	25 (64,1)	0.71 (NS) (Pearson's chi-square)
	R-	2 (28,6)	5 (71,4)	
**EBV-STs**	Mean	46,1	24,3	p=0.25 (t-test)
	Standard deviation	68,2	58,2	
	Median	4	0,5	p=0.19 (Mann-Whitney U-test)
	Interquartile range	79	22	

Abbreviations: CMV, cytomegalovirus; EBV, Epstein Barr virus; D, donor; R, recipient; NS, non-significant.

Supplementary figures

Figure S1

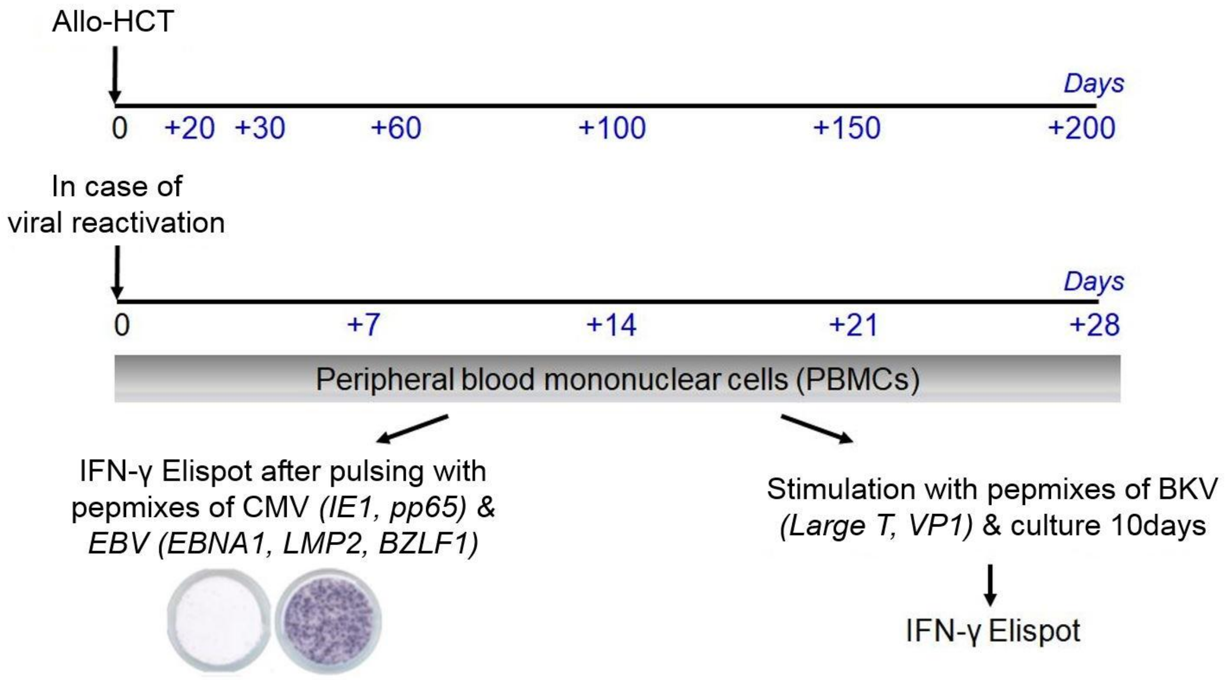

Figure S1. Design of the study. Immunological monitoring was performed on peripheral blood samples collected at days +20,+30,+60,+100,+150,+200 post allo-HSCT and in case of viral reactivation, weekly for one month. CMV- and EBV-STs were measured directly in blood, while BKV-STs were measured following 10-day in vitro expansion in culture, by using IFN-γ Elispot. PBMCs or expanded BKV-STs were pulsed with viral peptides (CMV: IE1, pp65; EBV: EBNA1, LMP2, BZLF1; BKV: Large T, VP1) and the secretion of IFN-γ was measured by Elispot assay. HCT, hematopoietic cell transplantation; PBMCs, peripheral blood mononuclear cells; CMV, cytomegalovirus; EBV, Epstein Barr virus; BKV, BK virus; IFN-γ, interferon-γ; CMV-STs, CMV-specific T cells; EBV-STs, EBV-specific T cells; BKV-STs, BKV-specific T cells.

Figure S2

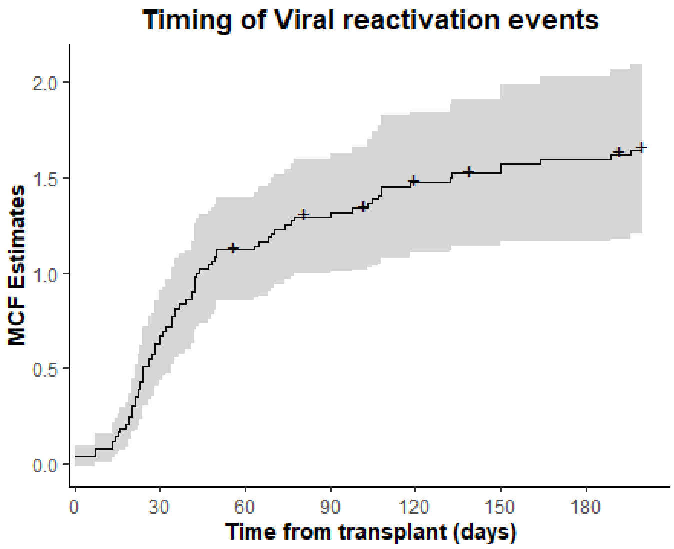

Figure S2. Timing of reactivation events for the whole cohort of patients. The nonparametric mean cumulative function (MCF) estimates represent the average number of events an individual had experienced during follow-up.

Figure S3

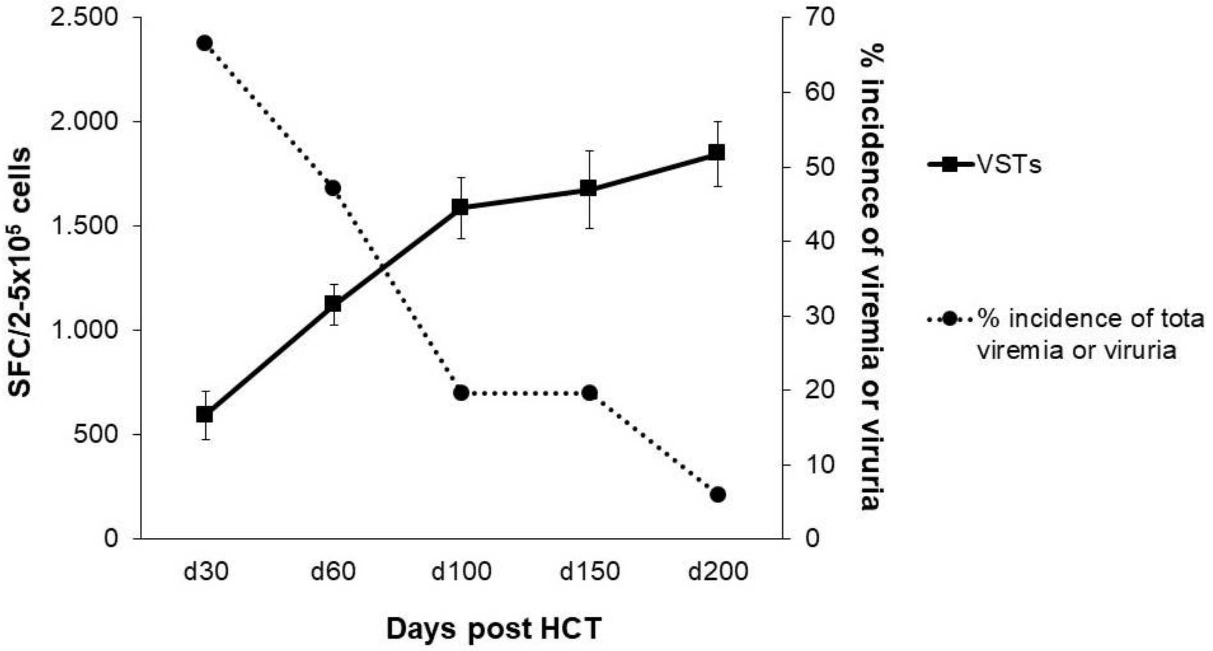

Figure S3. Inverse correlation between VSTs reconstitution (continuous line) **and incidence of viral reactivations** (dotted line)**.** VSTs, virus-specific T cells; SFC, spot-forming cells, HSCT, hematopoietic stem cell transplantation.

Figure S4

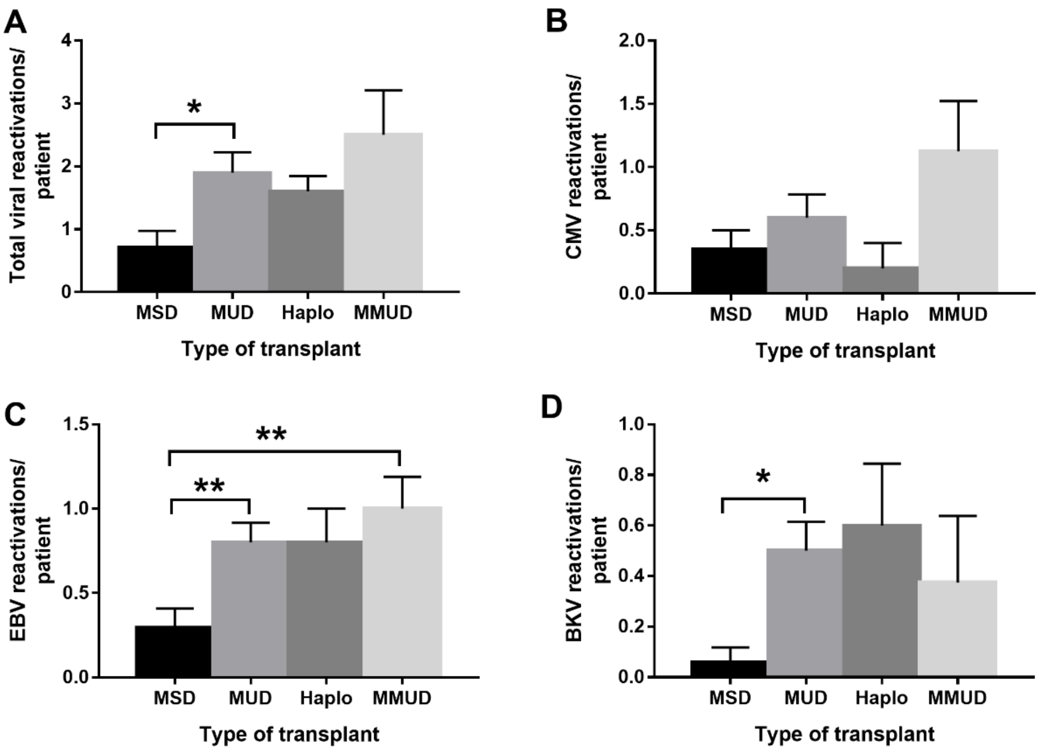

Figure S4. Viral reactivations per donor group. Bars depict the average number of total viral reactivations (A), reactivations by CMV (B), EBV (C) and BKV (D) per patient, grouped by the type of transplant. Lines represent the standard error of the mean. Differences between data sets were analyzed using one-way analysis of variance (ANOVA) followed by Tukey's comparisons test (*p≤0.037; **p=0.0048). CMV, cytomegalovirus; EBV, Epstein Barr virus; BKV, BK virus; MSD, matched sibling donor; MUD, matched unrelated donor; Haplo, haploidentical donor; MMUD, mismatched unrelated donor.

Figure S5

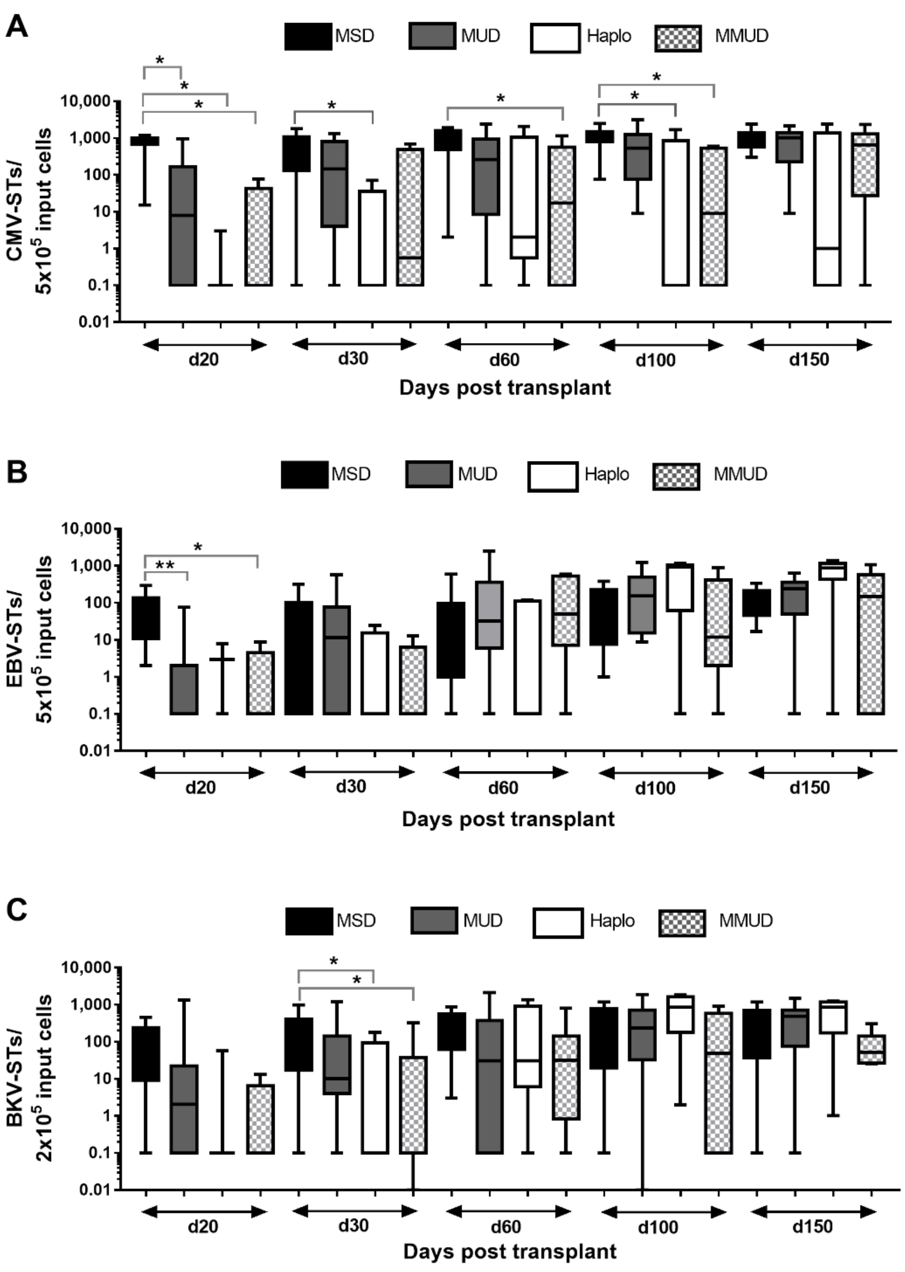

Figure S5. Kinetics of VST reconstitution post allo-HCT and per donor group (A-C). Bars represent the mean value within each group and error bars are the standard error of the mean. Differences between data sets were analyzed using Kruskal–Wallis one-way analysis of variance (ANOVA) (*p<0.05 and **p<0.0026). SFC, spot-forming cells; CMV-STs, CMV-specific T cells; EBV-STs, EBV-specific T cells; BKV-STs, BKV-

specific T cells; d, days post-transplant; MSD, matched sibling donor; MUD, matched unrelated donor; Haplo, haploidentical donor; MMUD, mismatched unrelated donor.

Figure S6

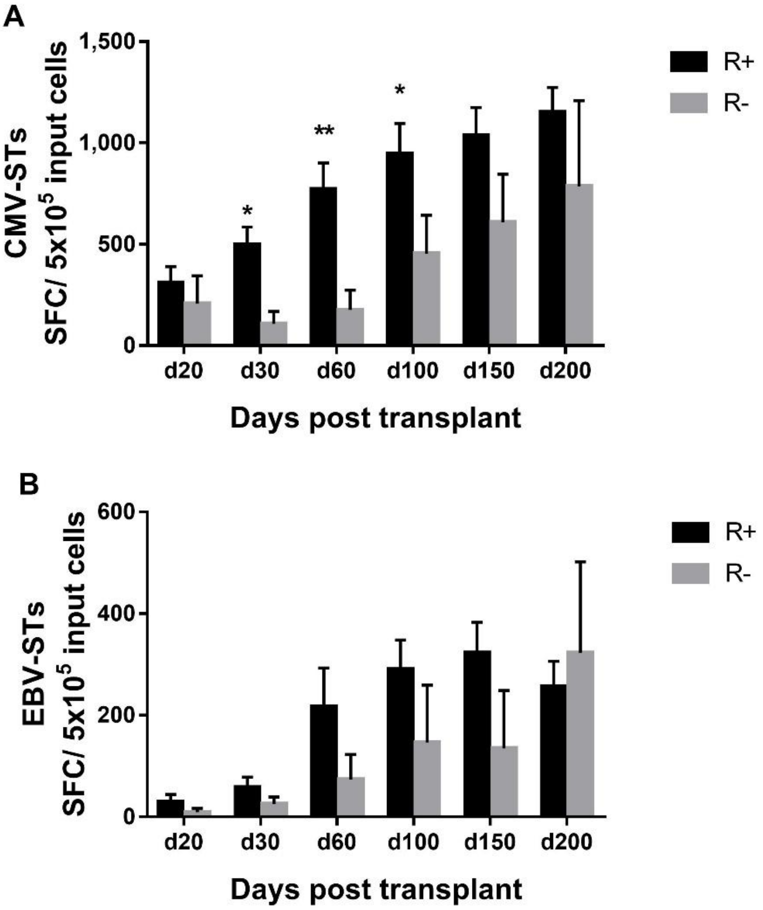

Figure S6. CMV- and EBV-ST reconstitution in seropositive vs seronegative recipients. Bars represent the mean value within each group and error bars are the standard error of the mean. Differences between data sets were analyzed using Mann-Whitney test (*p<0.026 and **p=0.03). SFC, spot-forming cells; CMV-STs, CMV-specific T cells; EBV-STs, EBV-specific T cells; R+, seropositive recipient; R-, seronegative recipient; d, days post transplant.

Figure S7

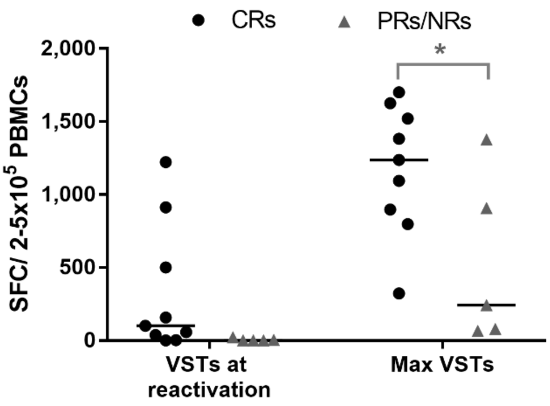

Figure S7. Virus-specific T cells in patients with viral disease. Baseline and maximum values of virus-specific T cells during reactivation in complete responders (CRs) or partial/ non responders (PRs/NRs). Each dot represents a patient. Horizontal lines represent the median value within each group. Differences between data sets were analyzed using unpaired t-test (*p=0.04). SFC, spot-forming cells; PBMCs, peripheral blood mononuclear cells; VSTs, virus-specific T cells.

Figure S8

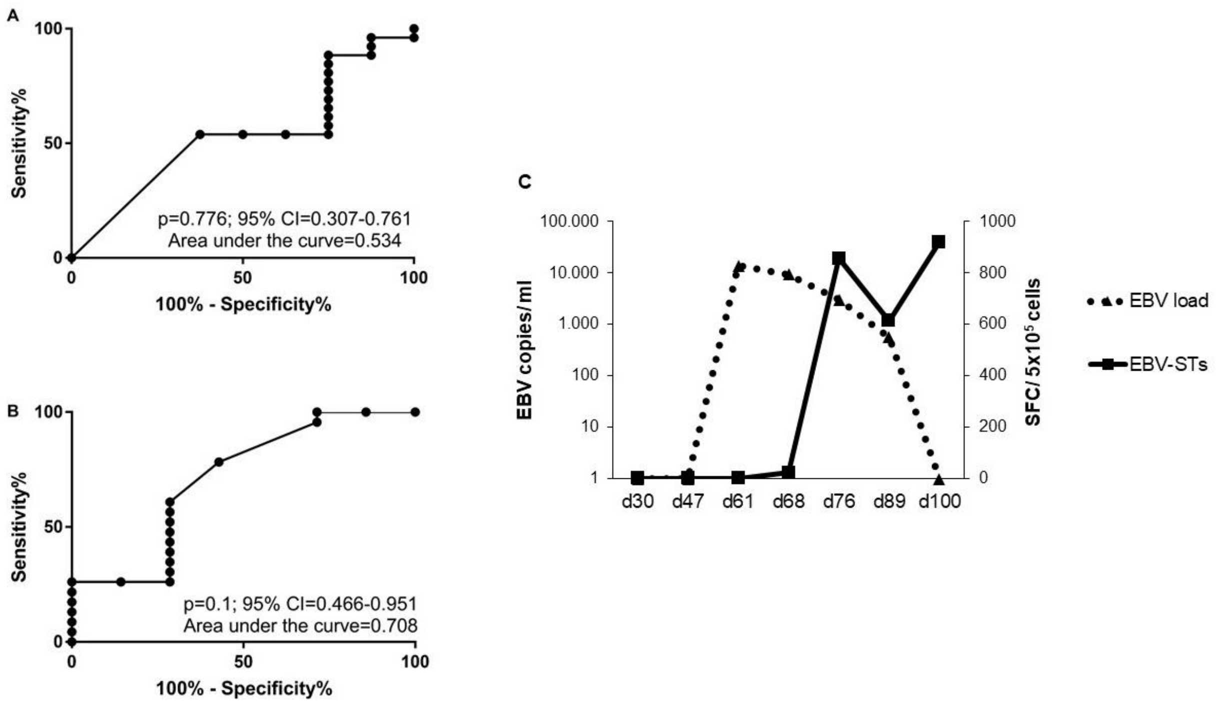

Figure S8. ROC curves of EBV-STs displaying as outcome complete response to reactivation (A-B) and a representative case of an EBV reactivating but untreated patient, on the basis of a substantial expansion of EBV-STs. EBV-STs, EBV-specific T cells.
